# Meeting report, ICH M7 relevant workshop: use of (Q)SAR systems and expert judgment

**DOI:** 10.1186/s41021-018-0107-2

**Published:** 2018-09-17

**Authors:** Masayuki Mishima, Tsuneo Hashizume, Yu Haranosono, Yusuke Nagato, Kentaro Takeshita, Junichi Fukuchi, Masamitsu Homma

**Affiliations:** 1 Research Division, Chugai Pharmaceutical Co., Ltd., Gotemba, Shizuoka, Japan; 2 Scientific Product Assessment Center, Japan Tobacco Inc., Yokohama, Kanagawa Japan; 3 Research & Development Division, Senju Pharmaceutical Co., Ltd., Kobe, Hyogo Japan; 4 Pharmaceutical & Healthcare Research Laboratories, FUJIFILM Corporation, Kaisei-machi, Ashigarakami-gun, Kanagawa Japan; 5 Safety Research Laboratories, Discovery and Research, Ono Pharmaceutical Co., Ltd., Sakai, Fukui, Japan; 6 Division of Pharmacopoeia and Standards for Drugs, Pharmaceuticals and Medical Devices Agency, Chiyoda-ku, Tokyo, Japan; 7 Division of Genetics and Mutagenesis, National Institute of Health Sciences, Kawasaki, Kanagawa Japan; 8 Fuji-Gotemba Research Laboratories, Chugai Pharmaceutical Co., Ltd., 1-135 Komakado, Gotemba, Shizuoka, 412-8513 Japan

**Keywords:** QSAR, In silico, Genotoxicity, Expert judgment, ICH M7

## Abstract

Use of Quantitative Structure-Activity Relationships ((Q)SAR) prediction tools has been increasing since the International Council for Harmonization of Technical Requirements for Pharmaceuticals for Human Use (ICH) M7 guideline was issued in June 2014. The Japanese Environmental Mutagen Society and the Bacterial Mutagenicity Study Group took the initiative of the workshop on (Q)SAR in 2016 to discuss using (Q)SAR to predict mutagenicity. The aim of the workshop was to form a common understanding on the current use of (Q)SAR tools in industry and for regulatory purposes and on the process of expert judgment. This report summarizes the general session that reviewed the use of (Q)SAR tools and the case study session that discussed expert judgment.

## Introduction

Because there is insufficient scientific consensus on the process for expert judgments made on results from Quantitative Structure-Activity Relationships ((Q)SAR) mutagenicity prediction tools, the Japanese Environmental Mutagen Society (JEMS) and the Bacterial Mutagenicity Study Group (BMS) took the initiative of the ICH M7 workshop held at the National Cancer Center in Tokyo in October, 2016 to discuss the procedure of expert judgment when evaluating Ames mutagenicity by (Q)SAR. Since the International Council for Harmonisation of Technical Requirements for Pharmaceuticals for Human Use (ICH) issued the ICH M7 guidelines “Assessment and control of DNA reactive (mutagenic) impurities in pharmaceuticals to limit potential carcinogenic risk” in June 2014, the use of (Q)SAR to evaluate mutagenic pharmaceutical impurities is expected to rapidly increase. Until then, many pharmaceutical companies had used (Q)SAR evaluation in-house as a tool to screen the toxicity of lead drug compounds or impurities, but the ICH-M7 guideline allows the (Q)SAR method to be used as a substitute for actual biological testing when evaluating mutagenicity for regulatory purposes. Consequently, there needs to be consensus among experts and appropriate guidance on its operation and the correct evaluation method. The ICH-M7 guideline recommends applying two complementary methods of (Q)SAR prediction in a (Q)SAR evaluation: one based on rulings by experts and one based on statistical information. If neither of the two complementary (Q)SAR methods produces an alert, the impurity can be judged to be of no mutagenic concern. However, if it is difficult to conclude easily because the predictions are conflicting or inconclusive, a final conclusion can be judged by an expert, but concrete procedures have not been shown specifically. In this workshop, more than a hundred experts of genetic toxicology and chemistry, mainly from the pharmaceutical industry and regulatory agencies, met together to develop a consensus on current standards for using (Q)SAR prediction tools and expert judgment.

## General session

At the beginning, three speakers talked about the current use of (Q)SAR since the publication of ICH-M7. Dr. Masamitsu Honma (National Institute of Health Science; NIHS) introduced the (Q)SAR tools that apply to ICH-M7. He is organizing an international collaborative project to improve the prediction power of (Q)SAR tools that are freely or commercially available in the world and presented the project progress and current perspective. Dr. Junichi Fukuchi (Pharmaceutical and Medical Device Agency; PMDA) outlined the Agency’s opinion on the use of (Q)SARs and how results should be interpreted, from the regulatory point of view, although PMDA has little experience of (Q)SAR evaluation as yet. Dr. Fukuchi also gave an example of similar structure search with the OECD toolbox functions. Dr. Masayuki Mishima (Chugai Pharmaceutical Co., Ltd.,) introduced the current extent of the use of (Q)SAR in pharmaceutical companies, based on a questionnaire survey on the use of (Q)SAR evaluation by Japanese Pharmaceutical Manufacturer’s Association (JPMA). There were 35 companies that perform (Q)SAR evaluation domestically, while one fifth of the companies were seeking for possibility of outsourcing (Q)SAR evaluation. A great majority of the companies used the two complementary types of tool: rule-based and statistic-based. The most popular tool was Derek, followed by CASE Ultra, Leadscope Model Applier (LSMA), Sarah, TOPKAT, and others. Because results from (Q)SAR tools were ambiguous, expert judgment was considered to be very important. In many companies, expert judgment was decided by toxicologists and chemists collaborating. However, it was not easy to overturn a positive prediction from (Q)SAR, probably because a consensus on the standard considerations/discussions for expert judgment has not yet been formed. Industry expected that the JEMS workshop, user meetings held by (Q)SAR vendors, and discussions by interested groups in JPMA would play important roles in a further understanding of expert judgment.

## Case study session

Several case studies were provided by the following speakers: Dr. Tsuneo Hashizume on N-acetyl-L-cysteine and 9-methylene-fluorene, Dr. Yu Haranosono on alternariol monomethyl ether (AME), Mr. Yusuke Nagato on 4-hydroxybutyl hydrogen sulfate, 2-amino-5-chlorobenzotrifluoride, and 2-(chloromethyl)pyridine hydrochloride, and Dr. Kentaro Takeshita on imidazole and an attempt to approach “not in domain” results. Table 1 shows the results from (Q)SAR tools with the expert judgments, which are the judgments given initially, before the meeting discussion.

**Table 1:**
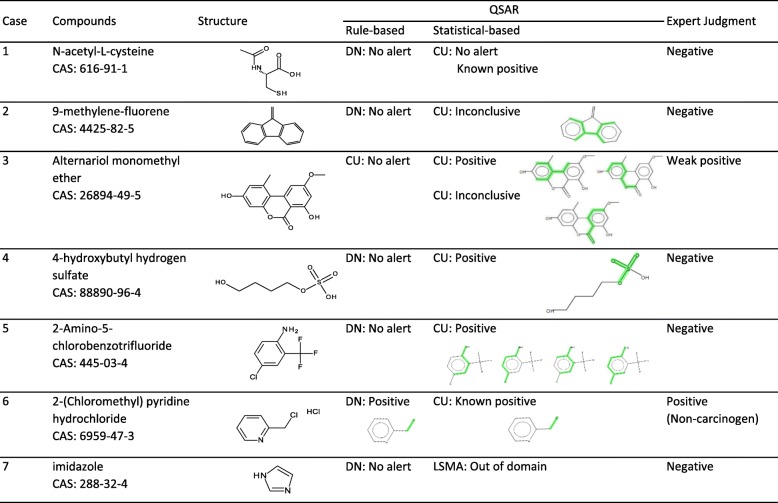
Summary of (Q)SAR and expert judgment

*DN* Derek Nexus, *CU* Case Ultra, *LSMA* Leadscope Model Applier

### Case study 1: N-acetyl-L-cysteine

Derek Nexus ver.4.1.0: Inactive.

CASE Ultra ver.1.5.2.0: Negative.

Expert judgment: Negative.

Rationale: The report by CASE Ultra included an Ames-positive reference by Glatt et al. [1], in which the dose-dependent and significant increase of mutant colonies was seen in TA97 in the presence of a metabolic activation system using rat kidney S9. The standard metabolic activation system in the Ames test is the rat liver S9 fraction, not the kidney. Stark et al. [2] have reported that glutathione and cysteine induced a positive response in the Ames test, and suggested that the mutagenic potential of thiol compounds was related to reactive oxygen species generated under the specific conditions used in the in vitro experiment, causing a false positive.

### Case study 2: 9-methylene-fluorene

Derek Nexus ver.4.1.0: Inactive.

CASE Ultra ver.1.5.2.0: Positive in GT1 A7B module. Negative in GT1 AT ECOLI module.

Expert judgment: Negative.

Rationale: The alert structure was planar (Table 1) but did not have any notable mutagenic structural alert, so the reference chemical structures for this alert were checked. The reference chemicals included some three-ring aromatic hydrocarbons, aromatic amines or amides, or heterocyclic hydrocarbons with epoxide, suggesting the alert structure found was a misleading substructure based on chemicals, other than the query chemical, that have mutagenic alerts.

### Case study 3: Alternariol monomethyl ether

CASE Ultra ver.1.6.0.3: Negative in GT_EXPERT.

CASE Ultra ver.1.6.0.3: Positive in GT1 A7B module, Inconclusive in GT1 AT ECOLI module.

Expert judgment: Weak positive.

**Fig. 1:**
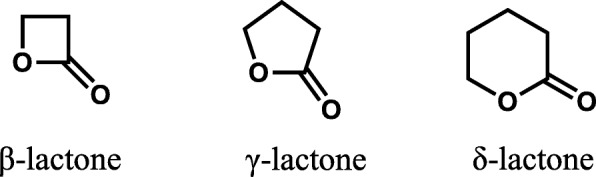
General structures of β-, γ-, and δ-lactone

**Fig. 2:**
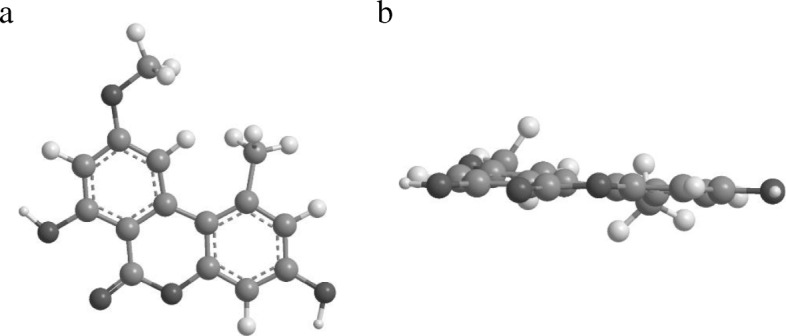
3D structure of AME generated by Chem3D®. **a**) front view, **b**) side-view. Gray: carbon, off-white: hydrogen, black: oxygen

Rationale: Other compounds with these structural alerts without quinone or quinolone (i.e. dihydroquercetin) were not mutagenic. Therefore, the structural alerts were rejected by the expert judge. However, AME has a δ-lactone part and a planar structure. The β-lactone (4-membered cyclic ester) reacts with primary or secondary amine to amide. The reactivity of lactone has the following order; β (4-membered) > γ (5-membered) > > δ (6-membered) (Fig. 1). The γ or δ lactones are not structural alerts but they have potential to react with amines at high concentration. AME has a planar structure that is an important feature for DNA intercalating. The planar region of AME has some substitution, i.e. methyl, methoxy, and hydroxyl groups (Fig. 2; drawn with Chem3D®, PerkinElmer Informatics Inc.). These substituted groups interrupt DNA intercalating because of their steric hindrance. The following mechanism was considered: 1) AME moves close to DNA and raises the local concentration of AME around DNA by “weak” intercalating; 2) δ-lactone of AME reacts with DNA to form covalent bonds. In conclusion, AME is classified as a “weak” DNA intercalator. This was in agreement with previous reports that AME was a weak mutagenic compound with or without metabolic activation in TA98 [3–5].

### Comment and discussion

At present, the planarity of chemicals is not routinely discussed because general (Q)SAR uses the 2D structure of chemicals. In future, some modeling software will enable the planarity to be evaluated in a qualitative manner. Experts should pay attention to the 3D structure of chemicals.

It was also reported that AME was non-mutagenic [6]. The purity of AME will differ in previous reports because AME is an extracted product from *Alternaria alternata*.

### Case study 4: 4-Hydroxybutyl hydrogen sulfate

Derek Nexus ver.4.1.0: Inactive.

CASE Ultra ver.1.5.2.0: Positive in GT1 A7B module. Negative in GT1 AT ECOLI module.

Expert judgment: Negative.

**Fig. 3:**
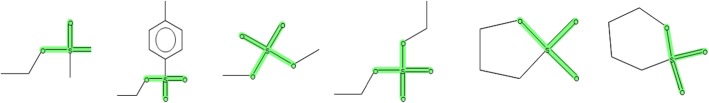
Training set compounds supporting alert fragments

**Fig. 4:**
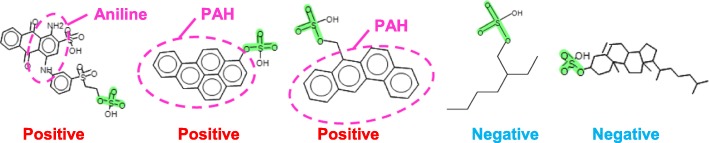
The mono-alkyl sulfate esters in the training set

Rationale: The mono-alkyl sulfate group was a major contribution to the positive prediction given by the statistical-based system. The training set compounds supporting the alert structure had other known structural alerts, such as alkyl sulfonate esters, dialkyl sulfates, or sultones (Fig. 3). As shown in Fig. 4, the Ames-positive mono-alkyl sulfates in the training set include other known mutagenic groups, such as aniline and polycyclic aromatic hydrocarbon (PAH). These well-known mutagenic groups were likely responsible for the Ames positive activity of the training set compounds but were not present in the query compound. It has been recognized that mono-alkyl sulfates are not electrophilic from their chemical reactivity, and they are consistently negative in the Ames assay [7, 8].

#### Comments and discussion

It is useful to review the training set compounds for other structure alerts. Other alert structures can be surveyed using Konsolidator of CASE Ultra.

### Case study 5: 2-Amino-5-chlorobenzotrifluoride

Derek Nexus ver.4.1.0: Inactive.

CASE Ultra ver.1.6.0.3: Positive in GT1 A7B and GT1 AT ECOLI module.

Expert judgment: Negative.

**Fig. 5:**
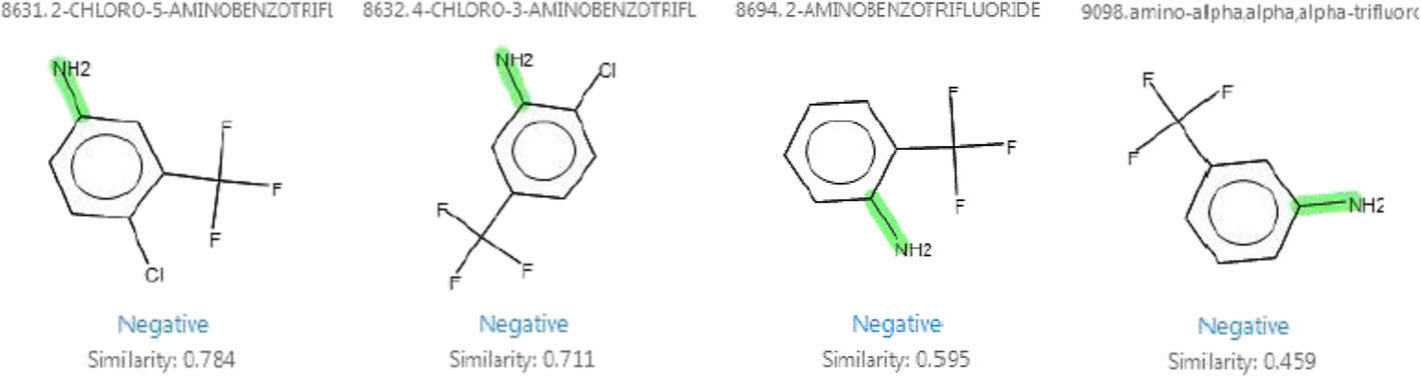
Structurally similar analogs of 2-amino-5-chlorobenzotrifluoride

**Fig. 6:**
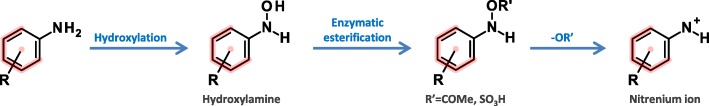
Metabolic activation pathway of aromatic amines

Rationale: The most significant contribution to the positive prediction was the primary aromatic amine group. All the structural analogs of the query compound found in a database were negative in the Ames assay (Fig. 5). Trifluoromethyl groups in the ortho position to the NH_2_ group of the aromatic amine strongly deactivate mutagenicity [6] that is caused by steric hindrance, which prevents metabolic activation to a hydroxylamine (Fig. 6). Additionally, electron-withdrawing groups have a resonance effect on the ring, thereby reducing electron density. This may disrupt the necessary metabolic step required to produce the mutagenic nitrenium ion (Fig. 6).

Ahlberg et al. [9] used SAR fingerprint analysis to show that the position and the type of an attached functional group contribute positively and negatively to aniline mutagenicity. Shamovsky et al. [10] suggested that there are three factors that make the aromatic amine mutagenic: (i) high affinity of the productive binding mode with CYP1A2 prior to proton abstraction, (ii) ease of proton abstraction from the NH_2_ group, and (iii) exothermicity of proton-assisted dissociation of hydroxylamine.

#### Comments and discussion

Metabolic activation of anilines starts with N-hydroxylation by CYP1A2 involving an initial proton abstraction from the NH_2_ group of anilines. The stability of anionic forms of anilines is significantly increased by para electron-withdrawing groups, such as the trifluoromethyl group and the fluoro group. In addition, stabilization of anionic forms of anilines is closely linked to metabolic activation of the nitrenium ion. As a result, the trifluoromethyl group or fluoro group in the para position activates the mutagenic potential of the anilines. The presence of “Strong deactivating” groups suggested that the compound was non-mutagenic but was insufficient evidence to support a negative conclusion. It is difficult to conclude that the compound is negative without Ames test results.

### Case study 6: 2-(Chloromethyl)pyridine hydrochloride

Derek Nexus ver.4.1.0: Positive.

CASE Ultra ver.1.6.0.3: Known positive in GT1 A7B. Negative in GT1 AT ECOLI module.

Expert judgment: Mutagenic non-carcinogen.

Rationale: The main contribution to the positive prediction was alkyl halide substructure (Table 1). The query compound was positive in the Ames test, an in vitro chromosomal aberration test, and an in vitro mouse lymphoma assay, but was negative in an in vivo micronucleus test [11–13]. The compound was not carcinogenic in animal carcinogenicity studies [14, 15].

#### Comments and discussion

Being Ames-positive but having a non-carcinogenic property is theoretically inconsistent. It may be recommended to add justifiable scientific evidence of the mechanism of non-carcinogenesis and to discuss the discrepancy between in vitro/ in vivo results.

### Case study 7: Imidazole

Derek Nexus ver. 5.0.2: Inactive.

LSMA ver. 2.0.3: Not in domain.

Expert judgment: Negative.

**Fig. 7:**
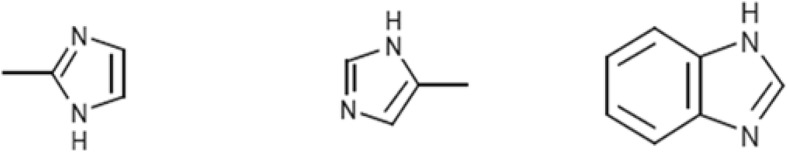
Ames negative “database analogs” with similar structures to imidazole

Rationale: Imidazole was negative in the Ames test [16] but the test was performed in 4 bacterial strains. Although no known or plausible DNA reactive group was identified by a visual inspection [17], additional data from Ames tests on compounds with similar structures to imidazole were used for further evaluation (Fig. 7). These structures were shown as “Database analogs” in LSMA. LSMA also showed these analogs were negative in the Ames tests. These results suggested that imidazole has a low risk of DNA reactivity.

#### Comments and discussion

The carcinogenicity data of structurally similar compounds should also be considered when the data of such compound were used to judge the risk of impurities, because there are some compounds which are not mutagenic but are carcinogenic.

### Case study 8: Attempt to provide support for out of domain structures, integrated partial structure assessment for out of domain structures

**Fig. 8:**
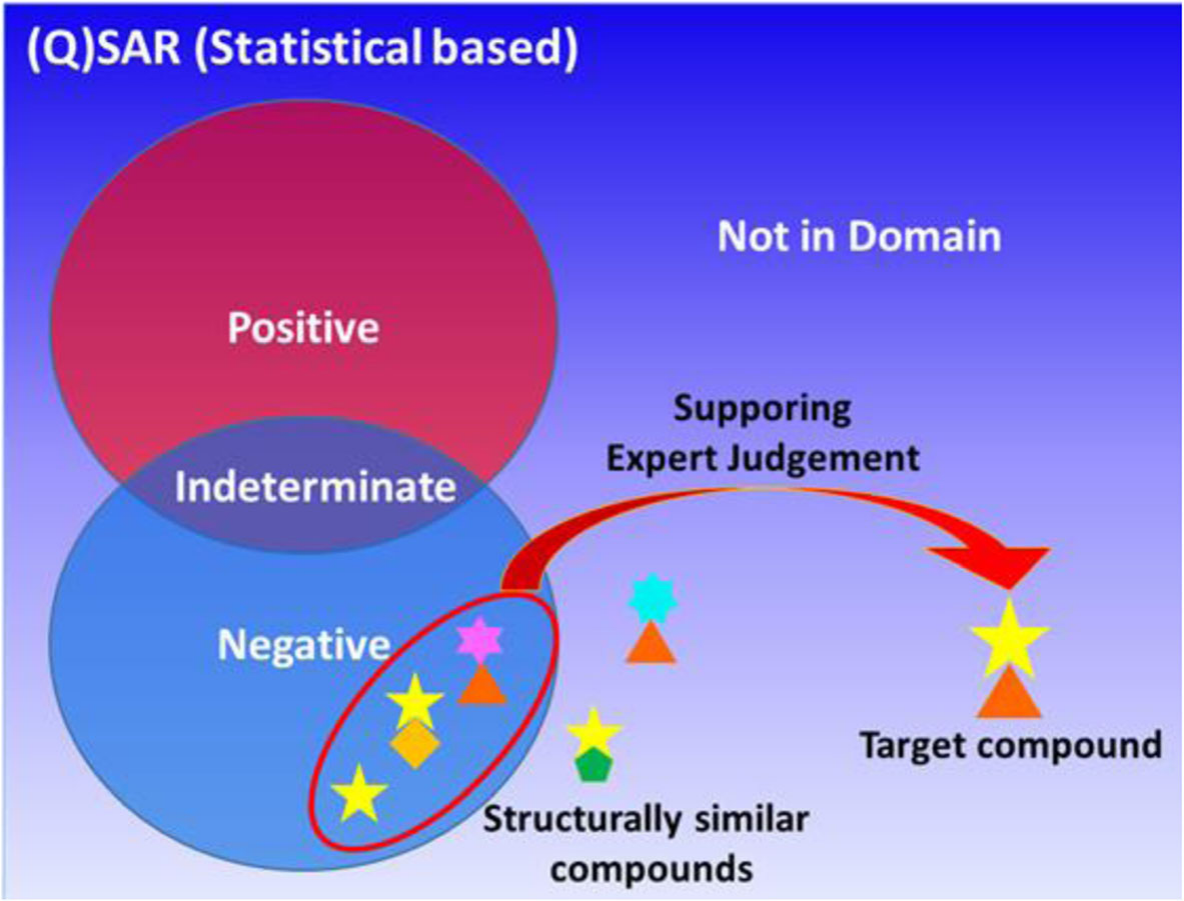
Concept of integrated partial structure assessment

**Fig. 9:**
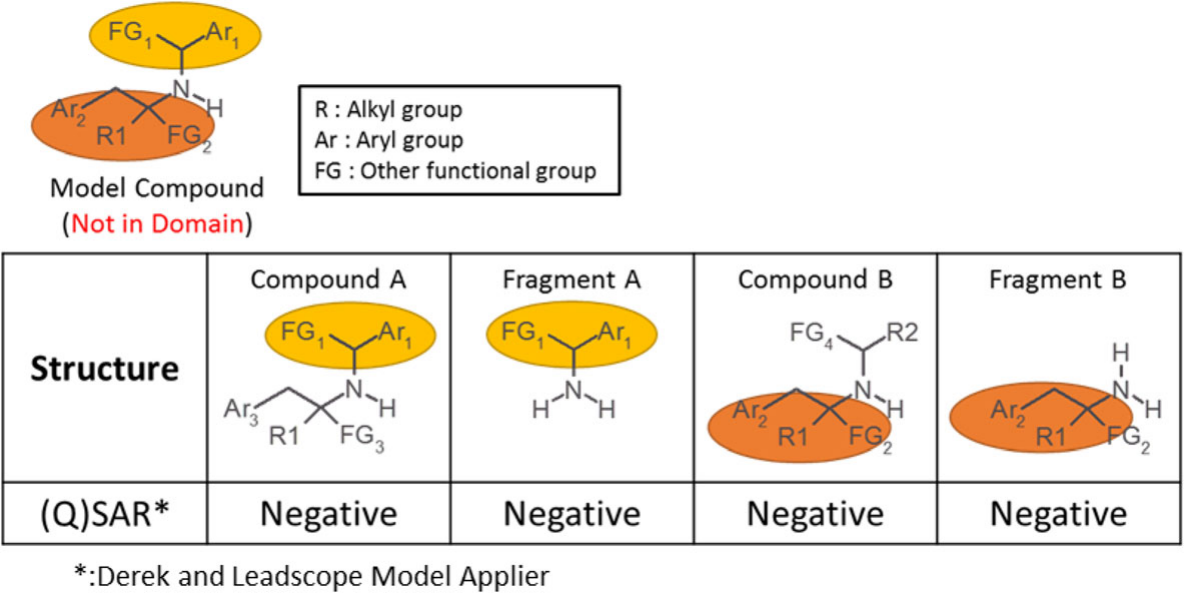
Representative structures predicted as negative in (Q)SAR

The concept of this attempt is represented in Fig. 8, which shows a compound that was judged by the (Q)SAR system as “not in domain”, but was assessed as Class 5 using integrated information of (Q)SAR results on partial structures similar to various parts of the query compound. An example is provided in Fig. 9. The model compound was predicted as “negative” by Derek, and “not in domain” by LSMA. This model compound contains two aryl groups, one alkyl group, and two other functional groups, but no known or plausible DNA-reactive groups were identified by visual inspection [17]. Structurally similar virtual compounds were examined by (Q)SAR. The virtual compounds were prepared by converting partial structures of the model compound with careful consideration of the following points to prevent getting a misleading negative.

1. Chemical reactivity
  The partial structure must be converted with little or no effect on reactivity. (e.g., conversion between isopropyl group and phenyl group). Mitigating factors (e.g., steric and electric effect) should also be considered.
2. Physicochemical property
  After conversion, the partial structure must not have a large increase in molecular weight, a large variation in molecular polarity, or a decrease in planarity.

Using structural fragments that have been disconnected from the original structure for the evaluation may be appropriate only if the disconnection does not reduce chemical reactivity [18]. Considering the points described above, structurally similar virtual compounds and structural fragments were prepared and evaluated (Fig. 9). Compound A includes the same structure as the upper part of the model compound. On the other hand, Compound B includes the same structure as the lower part of the model compound. These compounds and fragments were predicted as “negative” by both (Q)SAR software. Taken together, the model compound was predicted to be negative.

#### Comment and discussion

It was a really interesting attempt. However, the concept should be further validated and experience needs to be accumulated before using this for safety assessment.

## Conclusion

Consensus was reached on several points that need to be considered in an expert judgment:

1. Check another alert structure in the training compounds supporting the positive prediction for the query compound.
2. Consider chemical reactivity, e.g. the influence of side chains on electron density in the toxicophore.
3. When a metabolite was predicted to be active, accessibility for an enzyme is important.
4. Accessibility to DNA, e.g. planar structure, should also be checked.

Further discussion is needed on:

1. Acceptance of expert judgment based on chemical reactivity. Chemical reactivity is not necessarily relevant to a negative Ames result.
2. The range of “similar structures” and how to find structural similarity.
3. The availability of the OECD toolbox to define similar compounds.

## Abbreviations

(Q)SAR: (Quantitative) Structure-Activity Relationships
AME: Alternariol monomethyl ether
BMS: Bacterial Mutagenicity Study Group
ICH: International Council for Harmonization of Technical Requirements for Pharmaceuticals for Human Use
JEMS: Japanese Environmental Mutagen Society
JPMA: Japanese Pharmaceutical Manufacturer’s Association
PMDA: Pharmaceutical and Medical Device Agency
